# Eligibility for marine omega-3 fatty acid supplementation after acute coronary syndromes

**DOI:** 10.1016/j.athplu.2024.09.002

**Published:** 2024-09-15

**Authors:** Cédric Follonier, Gabriel Rabassa, Mattia Branca, David Carballo, Konstantinos Koskinas, Dik Heg, David Nanchen, Lorenz Räber, Roland Klingenberg, Moa Lina Haller, Sebastian Carballo, Stephan Windecker, Christian M. Matter, Nicolas Rodondi, François Mach, Baris Gencer

**Affiliations:** a Faculty of Medicine, University of Geneva, Switzerland; b Division of Cardiology, Geneva University Hospitals, Switzerland; c Department of Clinical Research, University of Bern, Switzerland; d Department of Cardiology, University Hospital of Bern, Switzerland; e Center for Primary Care and Public Health (Unisanté), University of Lausanne, Lausanne, Switzerland; f Department of Cardiology, Bern University Hospital and University of Bern, Bern, Switzerland; g Department of Cardiology, University Heart Center, University of Zurich, Switzerland; h Institute of Primary Health Care (BIHAM), University of Bern, Bern, Switzerland; i Department of General Internal Medicine, Geneva University Hospitals, Geneva, Switzerland; j Department of General Internal Medicine, Inselspital, Bern University Hospital, Bern, Switzerland; k Service of Cardiology, Lausanne University Hospital, Lausanne, Switzerland

**Keywords:** Acute coronary syndrome, Omega-3 fatty acids, Secondary prevention, Heart disease risk factor, Hypertriglyceridemia, Dyslipidemia

## Abstract

**Background and aims:**

The 2019 European Society of Cardiology guidelines for the management of dyslipidemia consider the use of high-dose marine omega-3 fatty acid (FA) eicosapentaenoic acid (EPA) supplementation (icosapent ethyl 2 × 2g/day) to lower residual cardiovascular risk in high-risk patients with hypertriglyceridemia. This study aimed to assess the eligibility for omega-3 FA-EPA supplementation in patients with acute coronary syndromes (ACS).

**Methods:**

In a prospective Swiss cohort of patients hospitalized for ACS, eligibility for marine omega-3 FA-EPA, defined as plasma triglyceride levels ranging from 1.5 to 5.6 mmol/l, was assessed at baseline and one-year follow-up and compared across subgroups. Lipid-lowering therapy intensification with statin and ezetimibe was modelled to simulate a hypothetical systematic treatment and its effect on omega-3 FA-EPA supplementation eligibility.

**Results:**

Of 2643 patients, 98 % were prescribed statin therapy at discharge, including 62 % at a high-intensity regimen; 93 % maintained it after one year, including 53 % at a high-intensity regimen. The use of ezetimibe was 3 % at discharge and 7 % at one year. Eligibility was observed in 32 % (32 % men, 29 % women) one year post-ACS. After modelling systematic treatment with statins, ezetimibe, and both, eligibility decreased to 31 %, 25 % and 24 %, respectively. Eligibility was higher in individuals aged <70 (34 vs 25 %), smokers (38 vs 28 %), diabetics (46 vs 29 %), hypertensive (35 vs 29 %), and obese patients (46 vs 22 % for normal weight), all with p-values <0.001.

**Conclusion:**

In a contemporary Swiss cohort of patients with ACS, up to 32 % would be eligible for omega-3 FA-EPA supplementation one year after ACS, highlighting an opportunity to mitigate residual cardiovascular risk in patients with ACS and hypertriglyceridemia.

## Highlights

• High-dose omega-3 fatty acid eicosapentaenoic acid (FA-EPA) supplementation has been shown to lower cardiovascular risk in high-risk patients with hypertriglyceridemia.
• Up to 1 in 3 patients were eligible for omega-3 FA-EPA supplementation one year after acute coronary syndromes.
• Eligibility for omega-3 FA-EPA is higher in those <70 years old and in smokers, diabetics, hypertensive, and obese patients.

## 1 Introduction

Patients with acute coronary syndromes (ACS) remain at high risk of recurrent major adverse cardiovascular events (MACE) despite lipid-lowering therapies such as statins [1]. Among markers of increased residual risk, elevated triglyceride levels are a potentially modifiable factor related to an increased risk of ischemic events. In a meta-analysis of triglyceride-lowering therapies including niacin, marine omega-3 fatty acid (FA) supplementations and fibrates, each reduction of triglycerides per 1 mmol/l was associated with a relative reduction of MACE by 16 % (95 % CI 6–25 %) [2].

In recent decades, there has been a growing focus on omega-3 fatty acids (FA) found in fish and seafood, namely eicosapentaenoic acid (EPA) and docosahexaenoic acid (DHA), due to their triglyceride-lowering properties. Additionally, circulating levels of omega-3 FA were inversely associated with the risk of cardiovascular and all-cause death [3,4]. Initial evidence from trials evaluating the efficacy of marine omega-3 FA supplementation for the prevention of MACE, generally at low doses (EPA ranging from 226 to 1800 mg/day) and in populations with modestly elevated triglycerides, was neutral, showing no benefit on cardiovascular risk reduction [5].

Compared to previous studies, the Reduction of Cardiovascular Events with Icosapent Ethyl-Intervention Trial (REDUCE-IT) showed a significant effect of high-dose omega-3 FA-EPA supplementation on clinical outcomes [6]. The REDUCE-IT study was a randomized, double-blind, placebo-controlled trial involving 8179 patients at high cardiovascular (CV) risk with fasting triglyceride levels between 1.52 and 5.63 mmol/l despite statin therapy. The use of 4 g of purified EPA (icosapent ethyl) was associated with a relative reduction of MACE by 25 % (95 % CI 17–32 %) compared with mineral oil placebo over a median follow-up of five years (p < 0.001). Following this new evidence, the 2019 European Society of Cardiology (ESC) guidelines for dyslipidemia recommended the use of marine omega-3 FA-EPA supplementation (2 × 2g/day of icosapent ethyl) to reduce residual cardiovascular risk in patients within the range of serum triglycerides investigated by the REDUCE-IT trial (class of recommendation IIa, level of evidence B) [1].

The proportion of patients who may have an indication for omega-3 FA-EPA supplementation after an ACS remains unknown. Eligibility for PCSK9 inhibitors after an ACS was previously reported in Switzerland [7]. Along the same lines, the current analysis aims to assess the eligibility for marine omega-3 FA-EPA supplementation in a Swiss cohort of patients with ACS according to the 2019 ESC guidelines, and better characterize the profile of patients eligible for omega-3 FA-EPA supplementation after ACS.

## 2 Methods

### 2.1 Study design, setting and sample

This study was conducted as part of the multidimensional prevention program after acute coronary syndrome (ELIPS, NCT01075867) project, a multicentric, observational, prospective cohort study, aiming to assess the quality of care and adherence to guidelines among patients with ACS discharged from four Swiss university hospitals (Bern, Geneva, Lausanne, and Zürich). ELIPS is a subproject of the Special Program University Medicine – Acute Coronary Syndrome (SPUM-ACS, NCT01000701) with extended data collection and follow-up. This study included ELIPS adult (≥18 years) participants hospitalized for ACS (definition provided in the **Supplementary Methods**) and discharged alive with triglyceride level assessments available at both baseline and one-year follow-up. Exclusion criteria were severe physical disability, dementia, or a life expectancy inferior to one year for non-cardiac reasons. The local ethics committees approved the study protocol, and all participants provided written informed consent.

Baseline demographic and medical data were collected at the time of hospital discharge by a team of trained study nurses. Subsequently, participants were invited to attend a clinical follow-up visit one year after inclusion, between January 1, 2009, and December 31, 2017. During the follow-up visit, information was collected on participation in a cardiac rehabilitation program and medication use. Blood samples were also obtained.

### 2.2 Lipid-lowering medications

Lipid-lowering medications were systematically assessed before hospital admission, at discharge for ACS, and at the one-year follow-up. The intensity of statin therapy was defined as low, moderate or high according to current lipid guidelines (Supplementary Table 1) [1]. During hospitalization for the ACS, physicians were encouraged to prescribe high-intensity lipid-lowering therapy following European guidelines, provided there were no specific contraindications for the individual participant [8,9]. During the one-year follow-up visit, participants were asked to bring their treatment list or pillboxes to confirm the prescribed treatment.

### 2.3 Lipid levels measurements

Baseline levels of total cholesterol, high-density lipoprotein cholesterol (HDL-C), and triglyceride were measured from the first available blood sample obtained within 24 hours following the hospital admission for ACS in participating centres. Lipid parameters were measured again at the one-year follow-up. They were measured at each participating site by a certified local laboratory. Low-density lipoprotein cholesterol (LDL-C) was calculated using the Friedewald equation.

### 2.4 Eligibility for marine omega-3 FA-EPA supplementation

The 2019 ESC guidelines specify that patients at high cardiovascular risk with elevation of plasma triglycerides (levels ranging from 1.5 to 5.6 mmol/) while already being treated with statins, are considered candidates for omega-3 FA-EPA supplementation [1]. Eligibility for omega-3 FA-EPA supplementation was defined in this study as triglyceride levels within the range proposed by the 2019 ESC guidelines on the management of dyslipidemias.

### 2.5 Statistical analysis

Baseline characteristics were compared between omega-3 FA-EPA supplementation eligible and non-eligible participants at baseline and after one year. Categorical variables were presented as frequencies and percentages and compared using the Chi-squared test or Fisher's exact test as appropriate; and continuous variables as mean with standard deviation (SD) or median with interquartile range (IQR) and compared with Student's t-test or Wilcoxon-Mann-Whitney *U* test. Eligibility for omega-3 FA-EPA supplementation was compared in predefined subgroups.

Observed eligibility for omega-3 FA-EPA supplementation was assessed in participants at baseline and one year after the index ACS. Moreover, eligibility was simulated under various therapeutic scenarios, considering systematic treatment with statin therapy, ezetimibe, and both, to reflect potential reductions of triglyceride levels due to these treatments. The methodology for the simulations is provided in the **Supplementary Methods**. A sensitivity analysis was conducted to assess the observed and simulated eligibility for omega-3 FA supplementation including participants with missing triglyceride values at baseline and/or one-year follow-up using multiple imputation. Multiple imputation of triglyceride values was performed using multivariate imputation by chained equations, using a truncated regression (lower bound greater than 0) and the baseline covariates as predictors for the imputation, producing 20 imputed datasets.

A significance level of p < 0.05 was used for all analyses. Analyses were performed using Stata statistical software, version 17.0.

## 3 Results

### 3.1 Baseline socio-demographic and medical characteristics

Between 2009 and 2018, 3762 individuals were discharged from four Swiss university hospitals after an ACS and were included in the cohort. Of these, 2643 completed a one-year follow-up with triglyceride levels available and were included in the present study (Supplementary Fig. 1). Baseline characteristics of included patients are described in Table 1. Participants were predominantly male (81 %) with a mean age of 61.4 years. Of them, 41 % were current smokers, 15 % were diabetic, 51 % had hypertension, and 12 % had experienced a previous myocardial infarction. Statin therapy was used in 25 % of participants at admission for ACS. The median LDL-C level was 3.2 mmol/l and triglyceride level was 1.2 mmol/l upon admission. At discharge, statin therapy was prescribed to 98 %, of participants with 62 % on a high-intensity regimen, and ezetimibe to 3 % (Supplementary Table 2). After one year, 93 % were taking statin therapy, with 53 % on a high-intensity regimen, and 7 % were taking ezetimibe (Table 2). Characteristics of included and excluded due to missing triglyceride levels at baseline and/or one-year follow-up are compared in Supplementary Table 3.

**Table 1:** Baseline characteristics by observed eligibility for omega-3 FA-EPA supplementation.

Baseline characteristics		Observed eligibility for omega-3 FA-EPA supplementation based on baseline triglyceride levels			Observed eligibility for omega-3 FA-EPA supplementation based on one-year triglyceride levels		
	Overall (N = 2643)	Eligible (N = 971)	Non-eligible (N = 1672)		Eligible (N = 841)	Non-eligible (N = 1802)	
	n/N (%)	n/N (%)	n/N (%)	p-value	n/N (%)	n/N (%)	p-value
Female sex	496/2643 (19 %)	145/971 (15 %)	351/1672 (21 %)	<0.001	146/841 (17 %)	350/1802 (19 %)	0.206
Age at inclusion	61.4 ± 12.0	59.0 ± 11.6	62.8 ± 12.0	<0.001	59.6 ± 11.6	62.3 ± 12.1	<0.001
Caucasian ethnicity	2515/2637 (95 %)	921/969 (95 %)	1594/1668 (96 %)	0.564	805/839 (96 %)	1710/1798 (95 %)	0.371
BMI (kg/m^2^)	27.1 ± 4.2	28.2 ± 4.3	26.5 ± 4.1	<0.001	28.3 ± 4.4	26.6 ± 4.0	<0.001
Completed high school or university	903/2587 (35 %)	316/946 (33 %)	587/1641 (36 %)	0.231	257 (31 %)	646 (37 %)	0.008
Cardiovascular risk factors							
*Current smoking*	1077/2643 (41 %)	453/971 (47 %)	624/1672 (37 %)	<0.001	405/841 (48 %)	672/1802 (37 %)	<0.001
*Diabetes mellitus*	407/2642 (15 %)	184/970 (19 %)	223/1672 (13 %)	<0.001	189/841 (22 %)	218/1801 (12 %)	<0.001
*Hypertension*	1341/2642 (51 %)	520/970 (54 %)	821/1672 (49 %)	0.026	466/841 (55 %)	875/1801 (49 %)	0.001
*Obesity (BMI >30 kg/m*^*2*^*)*	541/2628 (21 %)	271/965 (28 %)	270/1663 (16 %)	<0.001	246/839 (29 %)	295/1789 (16 %)	<0.001
Medical history at inclusion							
Myocardial infarction	321/2640 (12 %)	136/970 (14 %)	185/1670 (11 %)	0.026	125/840 (15 %)	196/1800 (11 %)	0.003
Coronary revascularization	399/2643 (15 %)	169/971 (17 %)	230/1672 (14 %)	0.012	151/841 (18 %)	248/1802 (14 %)	0.005
Stroke	53/2641 (2 %)	17/970 (2 %)	36/1671 (2 %)	0.478	14/841 (2 %)	39/1800 (2 %)	0.391
*Peripheral artery disease*	111/2642 (4 %)	47/970 (5 %)	64/1672 (4 %)	0.227	55/841 (7 %)	56/1801 (3 %)	<0.001
ACS diagnosis							
*STEMI*	1449/2642 (55 %)	484/970 (50 %)	965/1672 (58 %)	<0.001	439/841 (52 %)	1010/1801 (56 %)	0.065
*NSTEMI*	1086/2642 (41 %)	443/970 (46 %)	643/1672 (38 %)	<0.001	365/841 (43 %)	721/1801 (40 %)	0.107
*Unstable angina*	107/2642 (4 %)	43/970 (4 %)	64/1672 (4 %)	0.474	37/841 (4 %)	70/1801 (4 %)	0.527
Management of ACS							
*Coronary revascularization (PCI or CABG)*	2467/2643 (93 %)	905/971 (93 %)	1562/1672 (93 %)	0.828	782/841 (93 %)	1685/1802 (94 %)	0.616
*Cardiac rehabilitation*	1890/2580 (73 %)	680/951 (72 %)	1210/1629 (74 %)	0.124	572/819 (70 %)	1318/1761 (75 %)	0.008

Categorical data are presented as counts with percentages and continuous as means with standard deviations. BMI was missing for 15 patients. Baseline values are presented, unless otherwise specified (current smoking and alcohol consumption in the past 12 months). *Abbreviations: BMI = body mass index, ACS = acute coronary syndrome, NSTEMI = non-ST elevation myocardial infarction, STEMI = ST elevation myocardial infarction, PCI = percutaneous coronary intervention, CABG = coronary artery bypass graft*.

**Table 2:** Lipid parameters and use of lipid-lowering therapies at admission for ACS and at one-year follow-up according to omega-3 FA-EPA supplementation eligibility.

Characteristics (at baseline under baseline eligibility, at one-year under one-year eligibility)	At baseline			At one-year follow-up		
	Observed eligibility for omega-3 FA-EPA supplementation based on baseline triglyceride levels			Observed eligibility for omega-3 FA-EPA supplementation based on one-year triglyceride levels		
	Eligible (N = 971)	Non-eligible (N = 1672)		Eligible (N = 841)	Non-eligible (N = 1802)	
			p-value			p-value
**Lipid parameters**						
Lipid parameters at baseline						
*LDL-C (mmol/l)*	3.4 (2.7; 4.1)	3.1 (2.4; 3.8)	<0.001	2.2 (1.6; 2.9)	2.0 (1.6; 2.4)	<0.001
Non-HDL-C (mmol/l)	2.0 (1.8; 2.4)	1.6 (1.4; 1.9)	<0.001	2.0 (1.8; 2.3)	1.7 (1.5; 2.0)	<0.001
*HDL-C (mmol/l)*	1.0 (0.8; 1.2)	1.2 (1.0; 1.5)	<0.001	1.1 (0.9; 1.2)	1.3 (1.1; 1.5)	<0.001
*Triglycerides (mmol/l)*	2.1 (1.7; 2.7)	0.9 (0.7; 1.2)	<0.001	2.0 (1.7; 2.6)	0.9 (0.7; 1.2)	<0.001
*Triglycerides groups*			<0.001			<0.001
<1.69 mmol/l	188 (19 %)	1640 (98 %)		216 (26 %)	1783 (99 %)	
1.7–2.24 mmol/l	382 (39 %)	0 (0 %)		315 (37 %)	0 (0 %)	
≥2.25 mmol/l	401 (41 %)	32 (2 %)		310 (37 %)	19 (1 %)	
**Lipid-lowering therapies**						
Statin therapy			0.531			<0.001
*No*	714/943 (76 %)	1256/1612 (78 %)	0.205	90/830 (11 %)	95/1760 (5 %)	<0.001
*Low*	19/943 (2 %)	36/1612 (2 %)	0.779	19/830 (2 %)	29/1760 (2 %)	0.276
*Moderate*	145/943 (15 %)	219/1612 (14 %)	0.218	310/830 (37 %)	673/1760 (38 %)	0.696
*High*	65/943 (7 %)	101/1612 (6 %)	0.561	411/830 (50 %)	963/1760 (55 %)	0.013
Non-statin lipid-lowering therapies						
*Ezetimibe*	30/967 (3 %)	42/1667 (3 %)	0.377	69/837 (8 %)	123/1799 (7 %)	0.196
*Niacin*	12/967 (1 %)	7/1667 (0 %)	0.028	2/837 (0 %)	1/1799 (0 %)	0.238
*Fibrate*	0/967 (0 %)	1/1667 (0 %)	1.000	7/837 (1 %)	8/1799 (0 %)	0.265
**Non-lipid lowering guideline-directed medical therapies**						
Beta-blockers	163 (17 %)	297 (18 %)	0.523	654 (78 %)	1366 (76 %)	0.257
*ACE inhibitors*	115 (12 %)	211 (13 %)	0.581	494 (59 %)	1068 (59 %)	0.832
*Angiotensin receptor blockers*	186 (19 %)	305 (18 %)	0.569	195 (23 %)	386 (21 %)	0.313
*Calcium channel blockers*	83 (9 %)	163 (10 %)	0.331	86 (10 %)	188 (10 %)	0.945
Aspirin	251 (26 %)	407 (24 %)	0.401	814 (97 %)	1756 (98 %)	0.516

Lipid parameters, lipid-lowering, and non-lipid-lowering guideline-directed therapy use are presented at baseline (under baseline eligibility) and at one-year follow-up (under one-year eligibility). Categorical data are presented as counts with percentages, and continuous as median with interquartile range. At baseline, LDL-C was missing for 13 patients and HDL-C for 7. At one year-follow-up, LDL-C was missing for 13 patients, non-HDL-C for 13, and HDL-C for 6. *Abbreviations: LDL-C low density lipoprotein cholesterol, non-HDL-C = non-high-density lipoprotein cholesterol, HDL-C = high density lipoprotein cholesterol*.

### 3.2 Eligibility for marine omega-3 supplementation

Eligibility for marine omega-3 FA-EPA supplementation, assessmed based on baseline and one-year post-ACS triglyceride levels, is detailed in Table 1. Upon admission, 37 % of patients were eligible. Eligible patients were younger (59.0 vs. 62.8 years, p < 0.001) and more likely to be male (85 vs. 79 %, p < 0.001), diabetic (19 vs 13 %, p < 0.001) obese (BMI >30 kg/m^2^, 28 vs 16 %, p < 0.001), hypertensive (54 vs 49 %, p = 0.026), and current smokers (47 vs. 37 %, p < 0.001). They also had higher LDL-C levels (3.4 vs. 3.1 mmol/l, p < 0.001) (Table 2).

After one year, 32 % of patients were eligible. Among the 971 patients eligible at baseline, 507 (52 %) were still eligible after one year despite widespread use of statin therapy and lifestyle optimization. The eligible group based on one-year triglyceride levels maintained a similar profile in terms of baseline characteristics, risk factors, and comorbidities compared to the eligible group based on baseline triglyceride levels (Table 1). Yet, the proportion of men in the eligible and non-eligible groups became similar (83 vs 81 %, p = 0.206). Additionally, eligible participants had less frequently taken part in cardiac rehabilitation (70 vs 75 %, p = 0.008). At one year follow-up, statin therapy was less frequent in eligible individuals (89 % vs. 95 %, p < 0.001), who also had higher plasma LDL-C (2.2 vs 2.0 mmol/l, p < 0.001) as shown in Table 2.

A sensitivity analysis, including participants with missing triglyceride levels at baseline and/or one-year follow-up, was performed, and yielded similar results (Supplementary Table 4).

### 3.3 Eligibility for marine omega-3 FA-EPA supplementation in patient subgroups

In the subgroup analysis, we found significant differences across key participants subgroups, based on baseline and one-year characteristics (Table 3). Younger individuals, aged less than 70 years, displayed higher eligibility at baseline (40 %) and at one year (34 %) compared to older individuals, of whom about a quarter were eligible at any time point, suggesting a strong association between younger age and eligibility at baseline and one-year follow-up (p < 0.001). While male sex was positively associated with eligibility at baseline (39 % of eligibility in males vs 29 % in females, p < 0.001), this association waned after one year (32 % eligibility in males vs 29 % in females, p = 0.206). No evidence of an association between educational level and eligibility at baseline was found (35 % eligibility in higher education vs 37 % in lower education, p = 0.224), but a positive association between eligibility and lower educational level was observed at one-year follow-up (29 % eligibility in higher education vs 34 % in lower education, p = 0.007).

**Table 3:** Observed eligibility for omega-3 FA-EPA supplementation in subgroups.

Characteristics	Observed eligibility for omega-3 FA-EPA supplementation based on baseline triglyceride levels			Observed eligibility for omega-3 FA-EPA supplementation based on one-year triglyceride levels		
	Eligible (N = 971)	Non-eligible (N = 1672)		Eligible (N = 841)	Non-eligible (N = 1802)	
	n/N (%)	n/N (%)	p-value	n/N (%)	n/N (%)	p-value
Age						
*≥70 years old*	185 (27 %)	491 (73 %)		171 (25 %)	505 (75 %)	
*<70 years old*	786 (40 %)	1181 (60 %)	<0.001	670 (34 %)	1297 (66 %)	<0.001
Sex						
*Women*	145 (29 %)	351 (71 %)		146 (29 %)	350 (71 %)	
*Men*	826 (39 %)	1321 (62 %)	<0.001	695 (32 %)	1452 (68 %)	0.206
Diabetes mellitus						
*Yes*	184 (45 %)	223 (55 %)		189 (46 %)	218 (54 %)	
*No*	786 (35 %)	1449 (65 %)	<0.001	652 (29 %)	1583 (71 %)	<0.001
Completed high school or University						
*Yes*	316 (35 %)	587 (65 %)		257 (29 %)	646 (72 %)	
*No*	630 (37 %)	1054 (63 %)	0.224	566 (34 %)	1118 (66 %)	0.007
Smoking						
*Yes*	453 (42 %)	624 (58 %)		405 (38 %)	672 (62 %)	
*No*	518 (33 %)	1048 (67 %)	<0.001	436 (28 %)	1130 (72 %)	<0.001
*Hypertension*			0.026			0.001
*Yes*	520 (39 %)	821 (61 %)		466 (35 %)	875 (65 %)	
*No*	450 (35 %)	851 (65 %)		375 (29 %)	926 (71 %)	
Previous myocardial infarction						
*Yes*	136 (42 %)	185 (58 %)		125 (39 %)	196 (61 %)	
*No*	834 (36 %)	1485 (64 %)	0.026	715 (31 %)	1604 (69 %)	0.003
Statin therapy, at baseline (under baseline eligibility), and one year (under one-year eligibility)						
*Yes*	255 (38 %)	414 (62 %)		749 (31 %)	1706 (70 %)	
*No*	714 (36 %)	1256 (64 %)	0.385	90 (49 %)	95 (51 %)	<0.001
BMI (kg/m2)						
*<25*	221 (26 %)	620 (74 %)		187 (22 %)	654 (78 %)	
*25-30*	473 (38 %)	773 (62 %)	<0.001	406 (33 %)	840 (67 %)	<0.001
*>30*	271 (50 %)	270 (50 %)	<0.001	246 (46 %)	295 (55 %)	<0.001
*Triglycerides at baseline (mmol/l)*						
<1.69	188 (10 %)	1640 (90 %)		404 (22 %)	1424 (78 %)	
1.7–2.24	382 (100 %)	0 (0 %)	<0.001	174 (46 %)	208 (54 %)	<0.001
≥2.25	401 (93 %)	32 (7 %)	<0.001	263 (61 %)	170 (39 %)	<0.001
Cardiac rehabilitation						
*Yes*	.	.		563 (30 %)	1290 (70 %)	
*No*	.	.	.	257 (36 %)	465 (64 %)	0.011
*Achieved LDL-C <1.*4 mmol/l *at one-year follow-up*						
*Yes*	.	.		709 (32 %)	1511 (68 %)	
*No*	.	.	.	132 (31 %)	291 (69 %)	0.820
*Achieved LDL-C <1.*8 mmol/l *at one-year follow-up*						
*Yes*	.	.		561 (34 %)	1086 (66 %)	
*No*	.	.	.	280 (28 %)	716 (72 %)	0.001

Categorical data are presented as counts with percentages, and continuous as means with standard deviations. BMI was missing for 15 participants. Baseline values are presented, unless otherwise specified. *Abbreviations: BMI = body mass index, LDL-C = low-density lipoprotein cholesterol*.

Furthermore, eligibility was more frequent in patients with cardiovascular risk factors such as diabetes mellitus (45 % vs 35 % in non-diabetics at baseline, p < 0.001, and 46 % vs 29 % in non-diabetics after one year, p < 0.001), smoking (42 % vs 33 % in non-smokers at baseline, p < 0.001, and 38 % vs 28 % in non-smokers after one year, p < 0.001) hypertension (39 % vs 35 % in non-hypertensive patients at baseline, p = 0.026, and 35 vs 29 % in non-hypertensive patients after one year, p = 0.001), and obesity defined as BMI >30 kg/m^2^ (50 % vs 26 % in those with BMI <25 kg/m^2^ at baseline, p < 0.001, and 46 % vs 22 % in those with BMI <25 kg/m^2^ after one year, p < 0.001).

Patients with statin therapy prescriptions at the one-year follow-up were less likely to be eligible at the one-year follow-up (31 vs 49 %, p < 0.001), as well as those who attended cardiac rehabilitation (30 vs 36 %, p = 0.011).

### 3.4 Effect of lipid-lowering therapies on eligibility for marine omega-3 FA-EPA supplementation

**Fig. 1:**
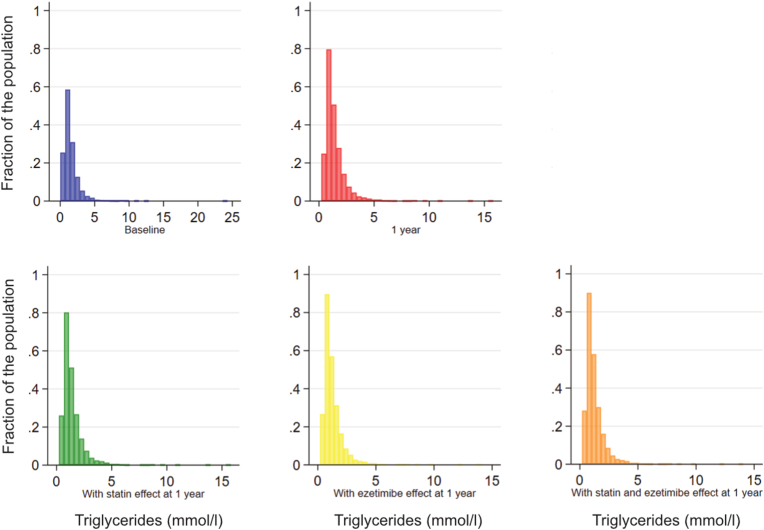
Distribution of observed and simulated triglyceride levels**.***Distribution of observed* triglyceride levels *at baseline (top left) and attheone-year follow-up (top right), and simulated considering systematic statin treatment attheone-year follow-up (bottom left, 15 % relative reduction oftriglyceride levelsin patients without statin), simulated considering systematic ezetimibe treatment attheone-year follow-up (bottom middle, 11 % relative reduction oftriglyceride levelsin participants without ezetimibe),andconsidering systematic statin and ezetimibe treatment attheone-year follow-up (bottom right, 11–24 % relative reduction oftriglyceride levels).*

Fig. 1 and Table 4 explore the eligibility for marine omega-3 FA-EPA supplementation under various scenarios, considering hypothetical systematic treatment with statin, ezetimibe, and both. At baseline, eligibility was observed in 37 % of participants. It decreased to 30 % assuming systematic use of statin therapy (scenario 1), 30 % assuming systematic use of ezetimibe (scenario 2) and 24 % assuming systematic use of both (scenario 3). After one year, eligibility was observed in 32 %. This proportion decreased to 31 % assuming systematic use of statin therapy (scenario 1), 25 % assuming systematic use of ezetimibe (scenario 2), and 24 % assuming systematic use of both (scenario 3).

**Table 4:** Observed and simulated eligibility for omega-3 FA-EPA supplementation.

	Eligibility for omega-3 FA-EPA supplementation
	n (%)
**Observed**	
Eligibility at baseline	971 (37 %)
Eligibility at the one-year follow-up	841 (32 %)
**Scenario 1: simulation considering systematic statin treatment (statin effect)**	
Eligibility at baseline	789 (30 %)
Eligibility at the one-year follow-up	810 (31 %)
**Scenario 2: simulation considering systematic ezetimibe treatment (ezetimibe effect)**	
Eligibility at baseline	799 (30 %)
Eligibility at the one-year follow-up	663 (25 %)
**Scenario 3: simulation considering systematic statin and ezetimibe treatment (statin and ezetimibe effect)**	
Eligibility at baseline	632 (24 %)
Eligibility at the one-year follow-up	644 (24 %)

Data are presented as counts with percentages. Scenario 1 assumes a systematic treatment with statins, scenario 2 a systematic treatment with ezetimibe, and scenario 3 a systematic treatment with a combination of both.

## 4 Discussion

In this large prospective cohort of post-ACS patients, one-third would be eligible for marine omega-3 FA-EPA supplementation according to the 2019 European guidelines on the management of dyslipidemias. The current findings bring important perspectives, given the possible wider use of high-dose omega-3 FA-EPA supplementation with icosapentethyl in clinical practice.

Despite the widespread implementation of statin therapy, where close to all patients were prescribed statin therapy at discharge and more than 90 % at the one-year follow-up, hypertriglyceridemia remained highly prevalent one year after ACS. Overall, 32 % were observed to be eligible for omega-3 FA-EPA (i.e., having triglycerides 1.5–5.6 mmol/l) one year after ACS, aligning with data from a large Swedish registry, where 40 % had triglycerides >1.4 mmol/l. In contrast, a French study using data from a national registry found that only 12.5 % of patients were eligible for omega-3 FA based on the REDUCE-IT criteria (including both triglycerides and LDL-C with statin therapy) [10]. This difference likely stems from the stricter criteria used in the French study compared to the ESC guidelines, which focus solely on triglycerides with statin therapy.

The observed eligibility for omega-3 FA-EPA was more pronounced in younger patients aged <70 years, and those presenting with traditional risk factors such as diabetes, smoking, and obesity. These findings reinforce the well-documented association between these characteristics and elevated triglyceride levels, emphasizing the importance of controlling them to lower residual cardiovascular risk associated with hypertriglyceridemia [[11], [12], [13], [14]]. For example, while smoking is linked with increased triglyceride levels, its cessation has been associated with a decrease intriglyceride levels. This information could be included in the comprehensive counseling provided to patients to further motivate them to quit smoking after ACS. Lower educational attainment, a key social determinant of health, was also associated with higher eligibility for omega-3 FA supplementation one year after ACS, reflecting higher triglyceride levels. This observation is expected, as individuals with lower educational attainment are more likely to adopt less-healthy lifestyle behaviors and have a higher prevalence of cardiovascular risk factors, including metabolic syndrome and diseases [[15], [16], [17], [18]]. Individuals with lower socioeconomic status also tend to have poorer control of cardiovascular risk factors and outcomes after ACS [19].

Regarding the effect of lipid-lowering therapies on the eligibility for omega-3 FA-EPA supplementation, a hypothetical systematic use of statin therapy yielded only a modest effect on the simulated eligibility, while a hypothetical systematic use of ezetimibe yielded a more pronounced effect. It stems from the limited use of ezetimibe in this cohort, probably because most of the participants were enrolled before the publication of the IMPROVE-IT results [20]. Among other classes of triglyceride-lowering agents, fibrates have demonstrated a class effect for reducing triglycerides, but market-available fibrates have not shown significant cardiovascular risk reduction in a meta-analysis of outcome trials [21]. This analysis further underscores the necessity for validated therapeutic approaches to effectively manage patients who continue to exhibit elevated triglyceride levels despite conventional lipid-lowering therapies.

Despite increasing interest in the role of omega-3 FA in preventing atherosclerotic cardiovascular disease, utilization of this treatment remains controversial [22,23]. Outcome trials, conducted in diverse populations with varying prevalences of secondary prevention and investigating the effects of different marine omega-3 FA derivatives (e.g., EPA, DHA, or their combinations) administered at various dosages, yielded heterogeneous results [24]. The recent REDUCE-IT stands out, demonstrating a 26 % relative risk reduction of MACE in participants receiving high-dose pure EPA, as opposed to a mineral oil placebo [6]. The subsequent randomized controlled trial STRENGTH examined the combined effect of EPA and DHA supplementation compared to a corn oil placebo and revealed no significant cardiovascular benefits, leading to premature discontinuation of the study [25]. The positive findings of REDUCE-IT and the neutral findings of STRENGTH have sparked controversy and a range of hypotheses explaining the pronounced effect of EPA in REDUCE-IT, including the use of pure EPA, high dosage (4g/day) and potential adverse effects of the mineral oil placebo. Indeed, unlike the participants receiving EPA, those receiving the mineral oil placebo had significantly higher levels of triglycerides, LDL-C, and hsCRP after two years compared to baseline. The possible detrimental effect of the REDUCE-IT placebo could potentially explain half of the effect size observed in the trial. To clarify this issue, a new trial comparing icosapent ethyl with corn oil, or even the STRENGTH trial's active arm, would be relevant [26]. The 2021 ESC guidelines on cardiovascular prevention downgraded the level of recommendation for using icosapent ethyl in patients with hypertriglyceridemia from IIa to IIb given those discrepancies [27]. However, both studies reported concordant effects concerning the increased risk of atrial fibrillation [28]. A subsequent meta-analysis realized after the publication of both trials' results indicated a significant reduction of MACE in participants with omega-3 FA-EPA supplementation, with a more pronounced effect in trials testing pure EPA (RR 0.72 [0.62–0.84]) in contrast to trials testing a combination of EPA and DHA (RR 0.92 [0.85–1.00]) [24]. The exact mechanisms of marine omega-3 FA-EPA in reducing cardiovascular events are still to be elucidated, but they may include a reduction in triglycerides, anti-inflammatory, and antithrombotic effects, and plaque stabilization and regression [[29], [30], [31]].

Multiple barriers may prevent implementation and adherence to high-dose omega-3 FA-EPA supplementation after ACS. At the healthcare system level, although high-dose omega-3 FA-EPA is available on the Swiss market, it is not yet covered by health insurance. At the healthcare provider and disease level, despite the REDUCE-IT results and subsequent meta-analyses, there is an ongoing debate within the cardiovascular community regarding the efficacy of this treatment and the relevance of hypertriglyceridemia. At the therapy level, the treatment requires taking two large capsules twice a day. These factors, combined with common adherence challenges such as health literacy and drug competition, pose significant obstacles [32].

While Omega-3 FA significantly reduced triglycerides in REDUCE-IT, they only slightly reduced cholesterol particles such as LDL-C and non-HDL-C, which are recognized causal markers in atherosclerotic cardiovascular disease. Novel hypertriglyceridemia treatments targeting messenger RNA are currently being evaluated and have shown promising results in phase 2 studies for reducing both triglyceride and non-HDL-C levels [33,34]. Results on clinical outcomes from phase 3 studies are awaited.

### 4.1 Strengths and limitations

This study is based on data from a large prospective cohort with adequate representativity of patients with ACS and robust adherence to lipid-lowering management guidelines. Its longitudinal design, featuring baseline and one-year follow-up lipid profile measurements, provides insights into the dynamic changes of lipid levels post-ACS. Furthermore, the study accounts for the potential enhancements of statin and ezetimibe therapies, aligning with the ever-improving adherence to lipid-lowering strategies.

The study faces some limitations. Approximately one-third of patients were excluded because of missing plasma triglyceride levels at baseline or one-year follow-up (e.g., follow-up interview carried out by phone); these patients were slightly older and more often female but had similar baseline triglyceride levels and lipid-lowering medication use at discharge compared to those included. Multiple imputation was used to address missing data in a sensitivity analysis, and the eligibility status remained comparable to the full case analysis. This suggests that their exclusion may not have substantially impacted the results. Additionally, slightly fewer women were included than in comparable European cohorts of patients with ACS. The results should therefore be generalized with more caution in older, predominantly female populations. The lack of data on regular omega-3 FA intake, whether through diet or supplements, constrains understanding of its effects on the lipid profiles. However, icosapent ethyl was not available on the Swiss market during the study period, and the rare use of fibrates was unlikely to interfere with the results. This limitation, coupled with a Swiss-based cohort, may restrict the generalizability of the findings to other populations with ACS and different demographic, lifestyle, and dietary characteristics.

## 5 Conclusion

In a contemporary Swiss cohort of individuals with ACS, up to 32 % of participants would be eligible for marine omega-3 FA-EPA supplementation one year after ACS according to the ESC guidelines. This high rate of eligibility may present an opportunity for mitigating residual cardiovascular risk in patients with hypertriglyceridemia. However, there remains an important gap to be addressed regarding the effectiveness and safety of omega-3 FA-EPA supplementation on long-term clinical outcomes.

## Conflict of interest

CMM has received research grants to the institution from the 10.13039/100000001Swiss National Science Foundation, 10.13039/100002129Swiss Heart Foundation, 10.13039/501100000691Swiss Academy of Medical Sciences, EliLilly, 10.13039/100004325AstraZeneca, 10.13039/100004337Roche, 10.13039/100002429Amgen, 10.13039/100004336Novartis, 10.13039/501100004191Novo Nordisk, and 10.13039/100030732MSD, including speaker or consultant fees. KK has received speaker fees/honoraria from 10.13039/100002429Amgen, 10.13039/100004339Sanofi, 10.13039/501100022274Daiichi Sankyo. LR has received research grants to institution by Abbott, Biotronik, Boston Scientific, Intraredx, Sanofi, Regeneron, Swiss Science Foundation, and speaker/consultation fees by Abbott, Amgen, Occlutech, Medtronic, Novo Nordisc, and Sanofi. SW reports research, travel or educational grants to the institution from Abbott, 10.13039/100020297Abiomed, 10.13039/100002429Amgen, Astra Zeneca, 10.13039/100004326Bayer, Bbraun, Biotronik, Boehringer Ingelheim, Boston Scientific, 10.13039/100008021Bristol Myers Squibb, 10.13039/100018599Cardinal Health, CardioValve, Cordis Medical, Corflow Therapeutics, 10.13039/100008322CSL Behring, 10.13039/501100022274Daiichi Sankyo, 10.13039/100006520Edwards Lifesciences, Farapulse Inc. Fumedica, Guerbet, Idorsia, Inari Medical, InfraRedx, Janssen-Cilag, Johnson & Johnson, Medalliance, Medicure, Medtronic, Merck Sharp & Dohm, Miracor Medical, Novartis, Novo Nordisk, Organon, OrPha Suisse, Pharming Tech. Pfizer, Polares, Regeneron, Sanofi-Aventis, Servier, Sinomed, Terumo, Vifor, and V-Wave. DC reports support to travel to/attend meetings. SW served as advisory board member and/or member of the steering/executive group of trials funded by Abbott, 10.13039/100020297Abiomed, 10.13039/100002429Amgen, Astra Zeneca, 10.13039/100004326Bayer, Boston Scientific, 10.13039/501100005035Biotronik, 10.13039/100008021Bristol Myers Squibb, 10.13039/100006520Edwards Lifesciences, 10.13039/501100023518MedAlliance, Medtronic, 10.13039/100004336Novartis, Polares, Recardio, Sinomed, 10.13039/501100008645Terumo, and V-Wave with payments to the institution but no personal payments. He is also member of the steering/executive committee group of several investigator-initiated trials that receive funding by industry without impact on his personal remuneration. DH is employed by the Department of Clinical Research, University of Bern, which has a staff policy of not accepting honoraria or consultancy fees. However, it is involved in the design, conduct, or analysis of clinical studies funded by not-for-profit and for-profit organizations. In particular, pharmaceutical and medical device companies provide direct funding to some of these studies. For an up-to-date list of conflicts of interest, see https://www.ctu.unibe.ch/research_projects/declaration_of_interest/index_eng.html. GR, CF, MB, DN, NR, FM, and BG have nothing to declare.

## Financial support

The SPUM-ACS consortium was supported by grants from the 10.13039/100000001Swiss National Science Foundation (project numbers 124112, 140336 and 163271) as well as from 10.13039/100016545Roche Diagnostics, Eli Lilly, AstraZeneca, Medtronic, 10.13039/100004334Merck Sharpe and Dome, 10.13039/100004337Roche, Sanofi-Aventis, and St. Jude Medical. Dr Gencer's research in cardiovascular prevention is supported by grants from the 10.13039/100000001Swiss National Science Foundation (project numbers 207881, 204361).

None of the funding institutions had any role in the design or execution of the study; in data collection, management, analysis, and interpretation; nor had any role in the preparation, review, or approval of the manuscript.
