# Supplementary material for: Eligibility for marine omega-3 fatty acid supplementation after acute coronary syndromes
Source: Atheroscler Plus. 2024 Sep 15;58:1–8. doi: 10.1016/j.athplu.2024.09.002 (PMC11439545; doi:10.1016/j.athplu.2024.09.002)
Supplement: Multimedia component 1 [file mmc1.docx]

**SUPPLEMENTARY MATERIAL**

**Tables of contents**

[Supplementary Methods 2](#_Toc170245648)

[Definitions of acute coronary syndromes 2](#_Toc170245649)

[Simulation of plasma triglyceride under various therapeutic scenarios 2](#_Toc170245650)

[Supplementary Figures 3](#_Toc170245651)

[Supplementary Figure 1. Study flowchart 3](#_Toc170245652)

[Supplementary Tables 4](#_Toc170245653)

[Supplementary Table 1. Classification of statin therapy intensity according to type and dose. 4](#_Toc170245654)

[Supplementary Table 2. Use of lipid-lowering therapies at discharge of hospitalization for index ACS according to marine omega-3 FA-EPA supplementation eligibility 5](#_Toc170245655)

[Supplementary Table 3. Characteristics of included and excluded patients 6](#_Toc170245656)

[Supplementary Table 4. Observed and simulated eligibility for omega-3 FA-EPA supplementation, including participants with missing triglyceride measure at baseline and/or one-year follow-up using multiple imputation (N = 3762) 8](#_Toc170245657)

[Supplementary References 9](#_Toc170245658)

# **Supplementary Methods**

## **Definitions of acute coronary syndromes**

Acute coronary syndromes (ACS) were defined as ST-elevation myocardial infarction, non-ST-elevation myocardial infarction and unstable angina, diagnosed within five days of symptoms onset. Inclusion required a diagnosis of ACS, defined by clinical presentations consistent with angina pectoris coupled with at least one of the following: (1) persistent ST-segment elevation or depression, T inversion or dynamic ECG changes, new left bundle branch block; (2) evidence of positive troponin by local laboratory reference values with a rise or fall in serial troponin levels; (3) known coronary artery disease, specified as status after myocardial infarction, coronary artery bypass graft, or percutaneous coronary intervention or newly documented ≥50% stenosis of an epicardial coronary artery during the initial catheterization.

## **Simulation of plasma triglyceride under various therapeutic scenarios**

Eligibility status for marine omega-3 FA-EPA supplementation was simulated under various therapeutic scenarios, considering systematic treatment with statin therapy, ezetimibe and both, to reflect potential reductions of TG due to these treatments. TG values were adjusted to simulate a systematic statin therapy by applying a fixed 15% relative reduction in participants not receiving statin therapy (denoted as "statin effect"). Similarly, TG values were adjusted to simulate systematic ezetimibe treatment by applying a fixed 11% relative reduction in those not receiving ezetimibe therapy (denoted as "ezetimibe effect"). Finally, a scenario was constructed where a systematic treatment with a combination of statin and ezetimibe was presumed, adjusting TG values to simulate the cumulative effect of statin and ezetimibe, applying a sequential combination of statin and ezetimibe relative reductions (i.e., 24.35%) of TG values in those on neither treatment, a 15% in those with ezetimibe but without statin, and an 11% in those with statin but without ezetimibe (denoted as "statin and ezetimibe effect").^1, 2^ These hypothetical simulations allowed the estimation of plasma TG under systematic treatment with statin and ezetimibe, thereby projecting eligibility for marine omega-3 FA-EPA supplementation under optimal treatment conditions.

# **Supplementary Figures**

## **Supplementary Figure 1.** Study flowchart


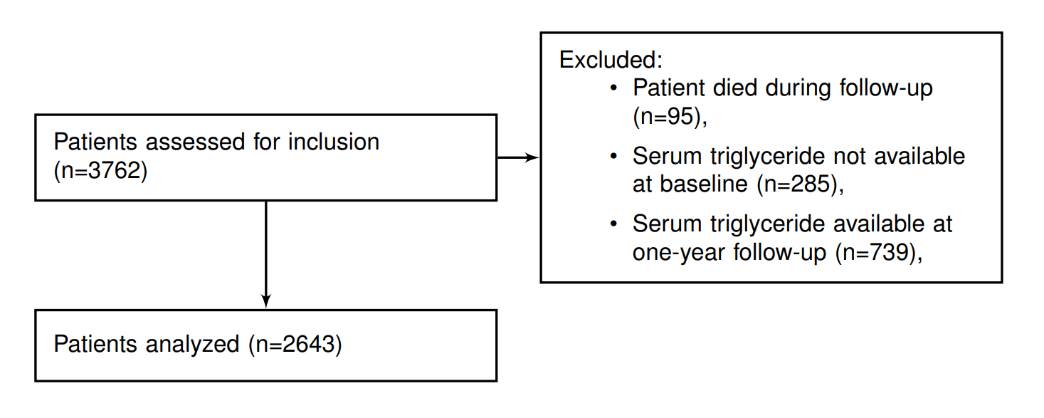


# **Supplementary Tables**

| **Supplementary Table 1**. Classification of statin therapy intensity according to type and dose. | |
| --- | --- |
| **Statin therapy intensity** | **Statin type and dose** |
| Low | Simvastatin 10 mg,  Pravastatin 10-20mg  Fluvastatin 20-40 mg |
| Moderate | Atorvastatin 10-20mg  Rosuvastatin 5-10 mg  Simvastatin 20-40 mg  Pravastatin 40-80 mg  Fluvastatin 80 mg |
| High | Atorvastatin 40-80mg  Rosuvastatin 20-40mg  Simvastatin 80mg |

| **Supplementary Table 2.** Use of lipid-lowering therapies at discharge of hospitalization for index ACS according to marine omega-3 FA-EPA supplementation eligibility | | | | | | | |
| --- | --- | --- | --- | --- | --- | --- | --- |
|  |  | **Observed eligibility for omega-3 FA-EPA supplementation based on baseline TG values** | | | **Observed eligibility for omega-3 FA-EPA**  **supplementation based on one-year TG values** | | |
| Characteristics | Overall  (N=2643) | Eligible  (N=971) | Non-eligible (N=1672) |  | Eligible  (N=841) | Non-eligible  (N=1802) |  |
|  | n/N (%) | n/N (%) | n/N (%) | p-value | n/N (%) | n/N (%) | p-value |
| Statin therapy at discharge |  |  |  | 0.452 |  |  | 0.606 |
| *No* | 41/2630 (2%) | 17/967 (2%) | 24/1663 (1%) | 0.530 | 16/838 (2%) | 25/1792 (1%) | 0.321 |
| *Low* | 22/ 2630 (1%) | 7/967 (1%) | 15/1663 (1%) | 0.629 | 9 /838 (1%) | 13/1792 (1%) | 0.360 |
| *Moderate* | 930/2630 (35%) | 325/967 (34%) | 605/1663 (36%) | 0.152 | 295/838 (35%) | 635/1792 (35%) | 0.908 |
| *High* | 1637/2630 (62%) | 618/967 (64%) | 1019/1663 (61%) | 0.179 | 518/838 (62%) | 1119/1792 (62%) | 0.756 |
| Non-statin lipid-lowering therapies at discharge |  |  |  |  |  |  |  |
| *Ezetimibe* | 70/ 2641 (3%) | 33/971 (3%) | 37/1670 (2%) | 0.068 | 34/840 (4%) | 36/1801 (2%) | 0.002 |
| *Niacin* | 3/ 2641 (0%) | 1/971 (0%) | 2/1670 (0%) | 1.000 | 1/840 (0%) | 2/1801 (0%) | 1.000 |
| *Fibrate* | 3/ 2641 (0%) | 0/971 (0%) | 3/1670 (0%) | 0.302 | 1/840 (0%) | 2/1801 (0%) | 1.000 |
| Data are presented as counts with percentages. | | | | | | | |

| **Supplementary Table 3.** Characteristics of included and excluded patients | | | |  |
| --- | --- | --- | --- | --- |
| Characteristics | Included  (N=2643) | Excluded due to missing TG values at baseline and/or one-year follow-up (N=844) |  | |
|  | n/N (%) | n/N (%) | p-value | |
| Female sex | 496/2643 (19%) | 224/844 (27%) | <0.001 | |
| Age at inclusion | 61.4 ± 12.0 | 63.5 ± 12.8 | <0.001 | |
| Caucasian ethnicity | 2515/2637 (95%) | 810/838 (97%) | 0.119 | |
| BMI (kg/m^2^) | 27.1 ± 4.2 | 26.8 ± 4.4 | 0.066 | |
| Completed high school or university | 903/2587 (35%) | 242/817 (30%) | 0.006 | |
| Cardiovascular risk factors |  |  |  | |
| Current smoking | 1077/2643 (41%) | 361/844 (43%) | 0.299 | |
| Diabetes mellitus | 407/2642 (15%) | 150/844 (18%) | 0.102 | |
| Family history of coronary artery disease (first degree relative, male relatives) | 727/2634 (28%) | 229/838 (27%) | 0.877 | |
| Hypertension | 1341/2642 (51%) | 472/844 (56%) | 0.009 | |
| Obesity (BMI >30 kg/m^2^) | 541/2628 (21%) | 161/835 (19%) | 0.429 | |
| Medical history at inclusion |  |  |  | |
| *Myocardial infarction* | 321/2640 (12%) | 135/844 (16%) | 0.004 | |
| *Coronary revascularization (PCI or CABG)* | 2467/2643 (93%) | 780/844 (92%) | 0.010 | |
| *Stroke* | 53/2641 (2%) | 27/844 (3%) | 0.044 | |
| *Peripheral artery disease* | 111/2642 (4%) | 55/844 (7%) | 0.009 | |
| ACS diagnosis |  |  | 0.446 | |
| *STEMI* | 107/2642 (4%) | 42/844 (5%) | 0.451 | |
| *NSTEMI* | 1086/2642 (41%) | 352/844 (42%) | 0.779 | |
| *Unstable angina* | 1449/2642 (55%) | 450/844 (53%) | 0.242 | |
| Management of ACS |  |  |  | |
| *Coronary revascularization (PCI or CABG)* | 399/2643 (15%) | 159/844 (19%) | 0.356 | |
| *Cardiac rehabilitation* | 1890/2580 (73%) | 527/778 (68%) | 0.003 | |
| Statin therapy at admission |  |  | 0.324 | |
| *No* | 1970/2555 (77%) | 597/806 (74%) | 0.079 | |
| *Low* | 55/2555 (2%) | 20/806 (2%) | 0.585 | |
| *Moderate* | 364/2555 (14%) | 134/806 (17%) | 0.099 | |
| *High* | 166/2555 (6%) | 55/806 (7%) | 0.745 | |
| Ezetimibe at admission | 72/2634 (3%) | 17/836 (2%) | 0.265 | |
| Statin therapy at one-year follow-up |  |  | <0.001 | |
| *No* | 185/2590 (7%) | 99/803 (12%) | <0.001 | |
| *Low* | 48/2590 (2%) | 16/803 (2%) | 0.800 | |
| *Moderate* | 983/2590 (38%) | 282/803 (35%) | 0.147 | |
| *High* | 1374/2590 (53%) | 406/803 (51%) | 0.217 | |
| Ezetimibe at one-year follow-up | 192/2636 (7%) | 49/832 (6%) | 0.168 | |
| Categorical data are presented as counts with percentages and continuous as means with standard deviations. BMI was missing for 24 patients, triglyceride for 350, LDL-C for 347, triglycerides for 339 and HDL-C for 340. Baseline values are presented, unless otherwise specified (active smoking and alcohol consumption in the past 12 months). *Abbreviations: BMI = body mass index, ACS = acute coronary syndrome, NSTEMI = non-ST elevation myocardial infarction, STEMI = ST elevation myocardial infarction, PCI = percutaneous coronary intervention, CABG = coronary artery bypass graft* | | | |  |

| **Supplementary Table 4.** Observed and simulated eligibility for omega-3 FA-EPA supplementation, including participants with missing triglyceride measure at baseline and/or one-year follow-up using multiple imputation (N = 3762) | |
| --- | --- |
|  | **Eligibility for omega-3 FA-EPA supplementation** |
|  | n (%) |
| **Observed** |  |
| Eligibility at baseline | 1390 (37%) |
| Eligibility at one-year follow-up | 1244 (33%) |
| **Scenario 1: simulation considering systematic statin treatment (statin effect)** |  |
| Eligibility at baseline | 1117 (30%) |
| Eligibility at one-year follow-up | 1187 (32%) |
| **Scenario 2: simulation considering systematic ezetimibe treatment (ezetimibe effect)** |  |
| Eligibility at baseline | 1128 (30%) |
| Eligibility at one-year follow-up | 947 (25%) |
| **Scenario 3: simulation considering systematic statin and ezetimibe treatment (statin and ezetimibe effect)** |  |
| Eligibility at baseline | 872 (23%) |
| Eligibility at one-year follow-up | 907 (24%) |
| Data are presented as counts with percentages. Scenario 1 assumes a systematic treatment with statins, scenario 2 a systematic treatment with ezetimibe, and scenario 3 a systematic treatment with a combination of both. | |

# **Supplementary References**

[1] Armitage, J, Baigent, C, Barnes, E, et al., Efficacy and safety of statin therapy in older people: a meta-analysis of individual participant data from 28 randomised controlled trials, The Lancet, 2019;393:407-415.

[2] Cannon, CP, Blazing, MA, Giugliano, RP, et al., Ezetimibe Added to Statin Therapy after Acute Coronary Syndromes, New England Journal of Medicine, 2015;372:2387-2397.
